# Enhanced visualization of the atrioventricular annulus using peak frequency and open-window mapping in right-sided accessory pathway ablation

**DOI:** 10.1016/j.hrcr.2025.08.028

**Published:** 2025-08-28

**Authors:** Kazuhisa Matsumoto, Kazuhiko Kuinose, Hitoshi Mori, Yoshifumi Ikeda, Ritsushi Kato

**Affiliations:** Department of Cardiology, Saitama Medical University International Medical Center, Hidaka-city, Saitama, Japan

**Keywords:** Peak frequency map, Open-window mapping, Accessory pathway, Catheter ablation, Atrioventricular reentrant tachycardia

**Key Teaching Points**

• Overlaying a peak frequency map onto an open-window map (OWM) enhances the delineation of the atrioventricular annulus.
• This combined mapping approach can aid in accurately identifying the atrial and ventricular insertion sites of accessory pathways.
• In cases where conventional OWM–guided ablation is unsuccessful, the Emphasis map may help guide effective ablation.

## Introduction

Accessory pathways (APs) are connections that directly establish electrical conduction between the atrial and ventricular myocardium. APs can have antegrade and/or retrograde conduction, sometimes leading to atrioventricular reentrant tachycardia (AVRT). When atrial fibrillation coexists, it can result in pseudo–ventricular tachycardia, which is known to be potentially life-threatening.^1^ Therefore, an electrophysiology study is recommended in patients with APs.^1^ Catheter ablation is an effective treatment for APs. Traditionally, point-by-point evaluation of local potentials was performed under fluoroscopic guidance. However, prolonged fluoroscopy exposure and the lack of contact force measurement made the procedure highly operator-dependent.^2^^,^^3^ Previous reports have shown a recurrence rate of ∼8%, indicating that a certain proportion of cases remain challenging to treat.^1^ In recent years, 3-dimensional mapping-guided ablation has become widely used. One recent advancement in mapping techniques is open-window mapping (OWM), which allows for the simultaneous inclusion of atrial and ventricular signals within the same window.^4^^,^^5^ OWM enables automatic annotation, reducing the need for manual adjustments in mapping. The OWM algorithm varies between vendors. In the EnSite system (Abbott, IL), annotation is automatically assigned at the maximum dV/dt, regardless of the 12-lead electrocardiogram morphology. In contrast, with CARTO (Johnson & Johnson, NJ), manual adjustment may be required depending on the waveform. OWM has several limitations. As with conventional AP mapping, mapping during tachycardia is preferable in order to avoid fusion with atrioventricular (AV) nodal conduction. In cases such as obliquely oriented APs where atrial-ventricular continuous potentials are difficult to record, visualization of the pathway can be challenging. Moreover, if the acquisition of the valvular annular potential is insufficient, there is a possibility of misinterpreting its anatomical evaluation. Currently, the EnSite X system uses Omnipolar Technology Near-Field (OTNF), which allows for the analysis of acquired potential frequencies to generate a peak frequency map. Overlaying the peak frequency map onto voltage maps or activation maps can help better highlight the regions of interest (Emphasis map). This mapping technique is commonly used to distinguish near-field from far-field potentials and to identify gaps in ablation lines. We report a case of a right-sided AP in which overlaying a peak frequency map onto the open-window map provided enhanced visualization of the annular region and was useful in guiding successful ablation.

## Case report

**Figure 1:**
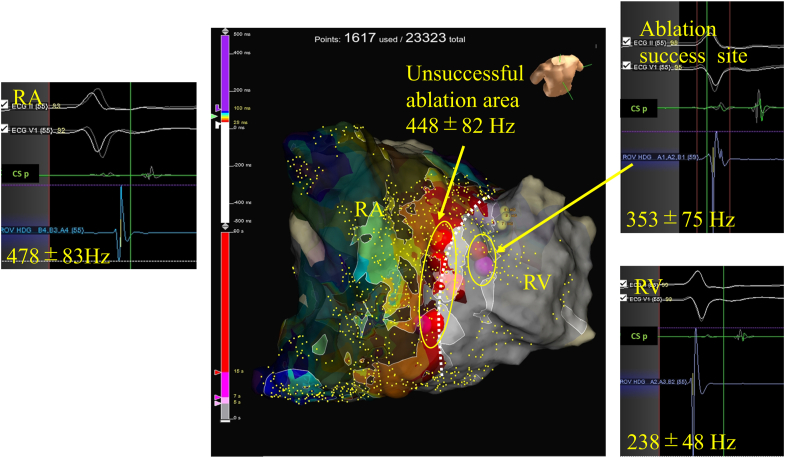
Emphasis map created by overlaying the peak frequency map onto the open-window map (OWM). The mean frequency of 20 randomly selected points in the right atrium (RA) was 478 ± 83 Hz, while in the right ventricle (RV), it was 238 ± 48 Hz, and the OWM boundary (*white dotted line*) had a mean frequency of 448 ± 82 Hz. The mean frequency of 20 points surrounding the successful ablation site (the boundary of the peak frequency map) was 353 ± 75 Hz.

**Figure 2:**
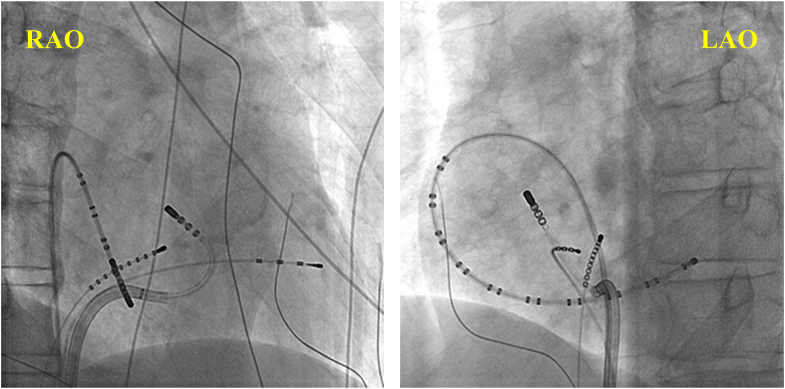
Fluoroscopic image showing catheter positioning during successful ablation. LAO = left anterior oblique; RAO = right anterior oblique.

A 60-year-old man was admitted to undergo an electrophysiology study and catheter ablation for recurrent narrow QRS tachycardia. No delta waves were observed on the baseline 12-lead electrocardiogram. The electrophysiology study revealed that retrograde conduction with the earliest atrial activation site on the right atrial lateral wall was observed during right ventricular pacing, without decremental properties. Atrial extrastimulation from the right atrium induced a short RP tachycardia without atrio-His interval prolongation. During tachycardia, a ventricular extrastimulus delivered within the effective refractory period) of the His advanced atrial activation. Ventricular overdrive pacing during tachycardia exhibited successful entrainment of atrial activation, and reinitiation of the tachycardia elicited a V-A-V pattern with a postpacing interval minus tachycardia cycle length of 70 ms. Differential atrial overdrive pacing confirmed ventricular-atrial (VA) linking, leading to the diagnosis of orthodromic AVRT mediated by a right-sided AP. OWM during tachycardia was created using an HD Grid catheter (Abbott, IL), revealing continuous potentials at the tricuspid annulus at the 12 o’clock position (Supplemental Video 1). Initially, we performed ablation guided by OWM on the atrial side of the tricuspid annulus, targeting the site with atrial-ventricular continuous potentials indicated by the white dotted line in Figure 1. Ablation was performed with a power setting of 50 W for 15 seconds, maintaining a contact force of 10–20 g using a TactiFlex SE catheter (Abbott, IL). To preserve consistency with the anatomical information provided by the OWM, ablation was conducted during tachycardia and ventricular pacing. This approach resulted in transient termination of the tachycardia, but VA conduction and tachycardia soon recurred. Therefore, we decided to further assess the annular region by superimposing an Emphasis map on the OWM. As shown in Figure 1, frequency analysis of 20 randomly selected points in the right atrium, right ventricle, and the boundary of the OWM revealed mean frequencies of 478 ± 83, 238 ± 48, and 448 ± 82 Hz, respectively. By setting the peak frequency cutoff to 350 Hz, the annular region could be effectively visualized. A frequency boundary was identified slightly more ventricular than the white dotted line. The mean frequency around the successful ablation site along the annulus was 353 ± 75 Hz (Figure 1). The successful ablation site could not be accessed from the atrial side because of poor catheter contact, so the catheter was inverted and approached from the ventricular side, resulting in successful ablation (Figure 2). The tachycardia was terminated immediately after energy delivery, and disappearance of VA conduction was confirmed (Supplemental Figure 1). To ensure complete elimination of the AP, we performed a 30-minute waiting period followed by an isoproterenol infusion and adenosine triphostate administration, confirming the absence of pathway reconduction.

## Discussion

We presented a case of a right-sided AP in which the tricuspid annulus was successfully visualized by overlaying the peak frequency onto the OWM. OWM, first described by Schricker et al,^4^ is a 3-dimensional electroanatomic mapping technique developed to facilitate the localization of APs in patients with AVRT. Unlike conventional mapping approaches that analyze atrial and ventricular signals separately, OWM incorporates both atrial and ventricular 12-lead electrocardiograms within a single mapping window. The annotation is placed at the site of the maximum absolute dV/dt, regardless of signal origin (atrial or ventricular). One of the key advantages of OWM is its ability to enhance visualization of the AV annulus. Simultaneous mapping of both chambers enables continuous analysis of conduction via the AP. However, delineation of the AV annulus necessitates manual analysis, and therefore operator expertise remains critical. OTNF analyzes the frequency of acquired potentials and highlights high-frequency regions, improving the clarity of target sites when combined with activation maps or voltage maps (Emphasis map). In the present case, setting the peak frequency threshold at 350 Hz allowed for clear delineation of the boundary between the atrium and the ventricle. OTNF allowed for delineation of the AV annulus based on the frequency differences between atrial and ventricular potentials. During tachycardia, the atrial side exhibited high frequencies ranging from 400 to 500 Hz, whereas the ventricular side had lower frequencies, ranging from 200 to 350 Hz. The difference in frequency may be attributed to variations in myocardial tissue characteristics, such as atrial or ventricular muscle properties, embryological origin, or the extent of myocardial electrical remodeling. Mayer et al^6^ evaluated the baseline peak frequency in patients undergoing ventricular tachycardia ablation and reported that in the ventricular myocardium with an amplitude of ≥1.5 mV, the peak frequency was ∼260 Hz, which is consistent with our findings. Further investigation with additional cases is needed to determine how frequency characteristics vary by location. Mizutani et al^7^ reported the use of OTNF-based peak frequency maps to highlight epicardial AP surrounding the coronary sinus at a 200 Hz setting. In this case, we also attempted to adjust the peak frequency settings to highlight the localization of the AP. Unfortunately, the atrial side exhibited uniformly high frequencies across both AP and non-AP regions, making it impossible to distinguish the AP site. Further investigation is warranted to determine the utility of the peak frequency map for the localization of APs.

## Conclusion

We demonstrated that the combination of OTNF-based peak frequency maps with OWM effectively visualized the valvular annulus, serving as an additional strategy for AP ablation.

## Disclosures

The authors have no conflicts of interest to disclose.
