# Supplementary figures and images for: Enhanced visualization of the atrioventricular annulus using peak frequency and open-window mapping in right-sided accessory pathway ablation
Source: HeartRhythm Case Rep. 2025 Aug 28;11(11):1216–8. doi: 10.1016/j.hrcr.2025.08.028 (PMC12666968; doi:10.1016/j.hrcr.2025.08.028)

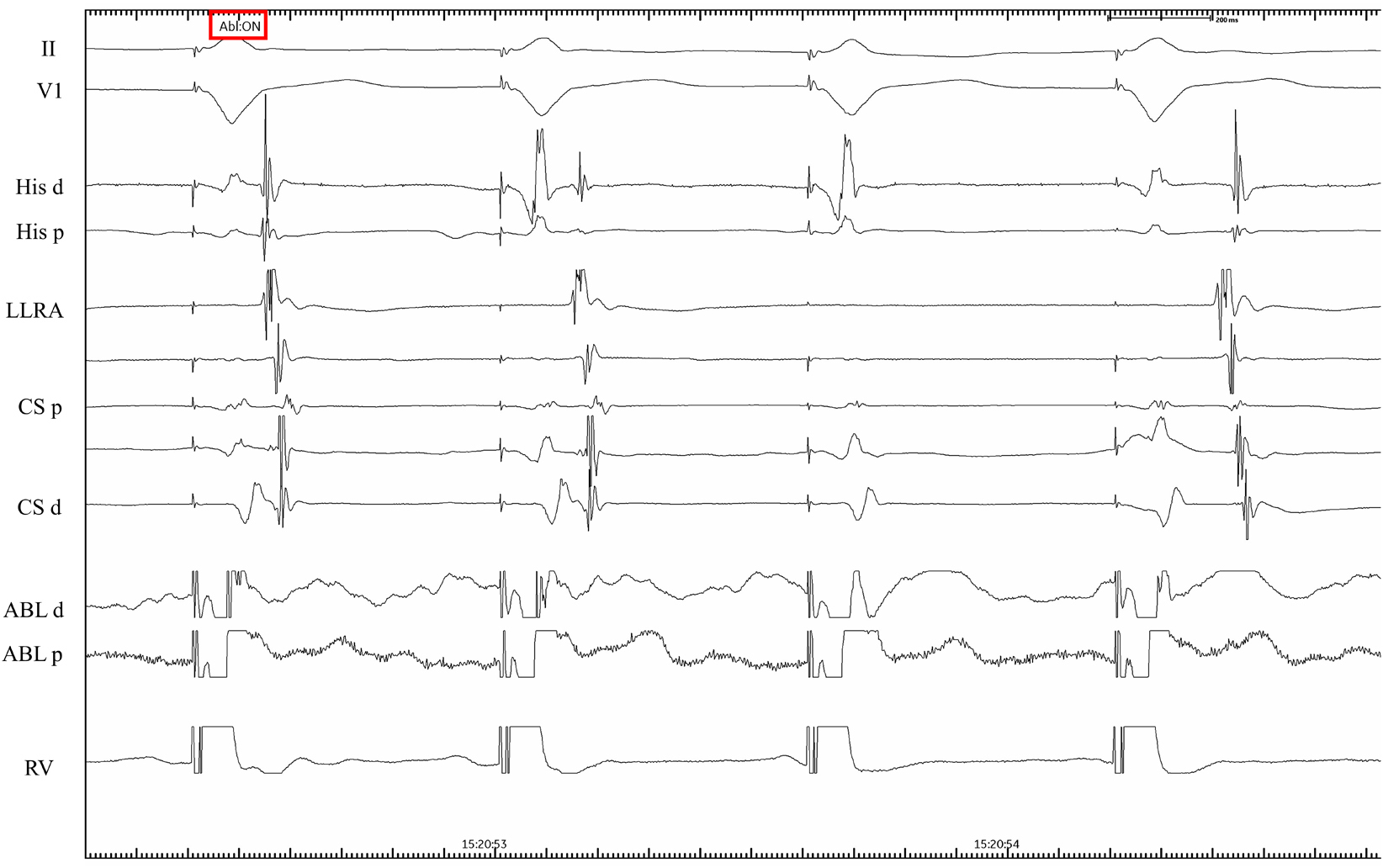

Supplement: Supplementary Figure 1 [file figs1.jpg]
