# Supplementary material for: Enhanced visualization of the atrioventricular annulus using peak frequency and open-window mapping in right-sided accessory pathway ablation
Source: HeartRhythm Case Rep. 2025 Aug 28;11(11):1216–8. doi: 10.1016/j.hrcr.2025.08.028 (PMC12666968; doi:10.1016/j.hrcr.2025.08.028)
Supplement: Supplementary Material [file mmc1.docx]

Supplemental figure 1

This figure shows the intracardiac electrogram at the time of successful ablation. VA dissociation occurred immediately after energy delivery.

Supplemental Video 1

An open-window map (OWM) created during tachycardia was superimposed with a Sparkle map. Although electrocardiograms appeared to radiate outward across the annular region identified by OWM, the exact localization of the accessory pathway remained unclear. By overlaying the Emphasis map, a frequency boundary was observed to shift slightly toward the ventricular side. This revealed a clear conduction pathway through the ablation success site, with activation propagating from the ventricular side to the atrium.
